# THE USE OF CAREGIVER SUPPORT SERVICES AND CAREGIVING IMPACT AMONG DIVERSE CAREGIVERS OF OLDER ADULTS WITH DEMENTIA

**DOI:** 10.1093/geroni/igad104.0361

**Published:** 2023-12-21

**Authors:** Erin Bouldin, Kaleb Eppich, Noah Webster, Amanda Leggett, Laura Zahodne

**Affiliations:** University of Utah, Salt Lake City, Utah, United States; University of Utah, Salt Lake City, Utah, United States; University of Michigan, Ann Arbor, Michigan, United States; Wayne State University, Detroit, Michigan, United States; University of Michigan, Ann Arbor, Michigan, United States

## Abstract

Caregiver support services may improve experiences among caregivers of people with dementia, but racial/ethnic disparities exist in service use. Using data from the 2021 National Study of Caregiving, we measured caregiver service use and caregiving impacts among 470 Black (n=187), Hispanic (n=37), and White (n=246) caregivers of older adults with possible/probable dementia from the National Health and Aging Trends Study. We classified caregivers as using at least one or none of the following services in the past year: caregiver support group, training, or respite care. We calculated the weighted proportion of caregivers reporting caregiver burden (at least one: physical, emotional or financial difficulty), exhaustion, and feeling overwhelmed within each racial/ethnic group. Caregiver service use varied by race/ethnicity (47% of Hispanic, 29% of Black, and 32% of White caregivers). Black and White caregiver service-users were significantly more likely to report a burden than their peers using no service (59% versus 36% of Black, p=0.04, and 90% versus 41% of White, p<0.001, caregivers). There was no significant difference in burden among Hispanic caregivers (59% versus 69%, p=0.65). In each racial/ethnic category, caregivers reported similar prevalence of exhaustion and feeling overwhelmed from caregiving whether they used a service or not. Based on these cross-sectional data, we hypothesize that Black and White caregivers who feel burdened use services more often, and that service use mitigates caregivers’ feelings of being exhausted or overwhelmed by caregiving, despite burden. Racial/ethnic differences highlight the need to understand contextual factors that influence caregiver burden and service use.

